# Supplementary material for: Prevalence of Untreated Early Childhood Caries of 5-Year-Old Children in Hong Kong: A Cross-Sectional Study
Source: Int J Environ Res Public Health. 2021 Nov 13;18(22):11934. doi: 10.3390/ijerph182211934 (PMC8625624; doi:10.3390/ijerph182211934)
Supplement: Supplementary file 1 [file ijerph-18-11934-s001.zip › ijerph-1447048-supplementary.pdf]

Supplementary Table S1. Published studies on ECC prevalence among 5-year-old children in Hong Kong.

**Year of survey**	**1986**	**1997**	**2007**	**2009**	**2016**
ECC prevalence	63%	44%	48%	49%	55%
Caries experience (dmft)	5.1	1.8	2.0	2.2	2.7
Untreated ECC prevalence of children with caries experience	-	> 90%	90%	> 95%	93%
Authors [Reference]	Wei et al [8]	Chu et al[9]	Lo et al[10]	Chu et al[11]	Chen et al[12]
Year of publication	1993	1999	2009	2012	2017

Supplementary 2. Questionnaire

Parental Questionnaire

Please answer all questions. All data and responses will be kept confidential and used only for research and study purposes. Thank you very much!

SECTION 1: Child's oral health-related behaviors

1. How often does your child brush his/her teeth each day?
   ☐ Less than once ☐ Once ☐ Twice or more

2. Does your child use toothpaste at present?
   ☐ No ☐ Yes

3. On average, how often does your child have a sweet snack between meals at present?
   ☐ Less than once a day ☐ Once a day ☐ Twice a day ☐ Three times or more a day

4. Who usually takes care of your child? (Please choose one)
   ☐ Parents ☐ Grandparents ☐ Domestic helper ☐ Others (e.g. relatives, friends)

5. Has your child ever seen a dentist?
   ☐ Yes, with regular review ☐ Yes, without regular review ☐ Never

SECTION 2: Child and family information

6. Name of child: __________________ Gender: ☐ Male ☐ Female
7. Contact tel. no: __________________
8. Date of birth: ____ / ____ / ______
   dd mm yyyy
9. Place of birth: ☐ Hong Kong ☐ Mainland China ☐ Others (please specify: _________)

10. Parent education level:

	Father	Mother
Junior secondary school (≤ 9 years)	☐	☐
Senior secondary school (10-13 years)	☐	☐
Post-secondary / university (≥ 14 years)	☐	☐

11. Average total monthly household income:
   ☐ Below $15,000
   ☐ $15,001 to $30,000
   ☐ Over $30,000

End of questionnaire.
Thank you for your kind participation.

家長問卷調查

請回答所有問題。所有答案及資料都會保密。謝謝！

<u>第一部份：子女的口腔衛生習慣</u>

1. 您的子女現時每日刷牙多少次？
   ☐ 少於一次　☐ 每天一次　☐ 每天兩次或以上

2. 您的子女刷牙時有沒有使用牙膏？
   ☐ 有　☐ 沒有

3. 您的子女每天於正餐以外進食了多少次零食？
   ☐ 少於一次　☐ 每天一次　☐ 每天兩次　☐ 每天三次或以上

4. 您的子女通常是由誰照顧？（請選其一）
   ☐ 父母　☐ 祖父母　☐ 家傭　☐ 其他（如親戚朋友）

5. 您的子女有沒有看過牙醫？
   ☐ 有，定期檢查　☐ 有，但不是定期檢查　☐ 沒有

<u>第二部份：子女個人及背景資料</u>

6. 子女姓名：＿＿＿＿＿＿＿＿＿＿　性別：☐ 男　☐ 女
7. 聯絡電話：＿＿＿＿＿＿＿＿＿＿
8. 出生日期：＿＿＿＿年＿＿＿月＿＿＿日
9. 出生地點：☐ 香港　☐ 中國內地　☐ 其他，請註明：
   ＿＿＿＿＿＿＿＿

10. 家長的教育程度：

	父	母
初中（在學9年或以下）	☐	☐
高中（在學10至13年）	☐	☐
大專或大學（在學14年或以上）	☐	☐

11. 家庭平均每月收入：
    ☐ $15,000 以下
    ☐ $15,001 至 $30,000
    ☐ $30,001 或 以上

問卷完

多謝你的參與
